# Novel strategies to reverse chemoresistance in colorectal cancer

**DOI:** 10.1002/cam4.5594

**Published:** 2023-01-16

**Authors:** Shu‐Chang Ma, Jia‐Qi Zhang, Tian‐Hua Yan, Ming‐Xing Miao, Ye‐Min Cao, Yong‐Bing Cao, Li‐Chao Zhang, Ling Li

**Affiliations:** ^1^ Institute of Vascular Disease, Shanghai TCM‐Integrated Hospital Shanghai University of Traditional Chinese Medicine Shanghai China; ^2^ Department of Physiology and Pharmacology China Pharmaceutic University Nanjing China; ^3^ Department of Pharmacy Shanghai Municipal Hospital of Traditional Chinese Medicine Shanghai China

**Keywords:** chemoresistance, colorectal cancer, novel strategies, reversal of resistance

## Abstract

Colorectal cancer (CRC) is a common gastrointestinal malignancy with high morbidity and fatality. Chemotherapy, as traditional therapy for CRC, has exerted well antitumor effect and greatly improved the survival of CRC patients. Nevertheless, chemoresistance is one of the major problems during chemotherapy for CRC and significantly limits the efficacy of the treatment and influences the prognosis of patients. To overcome chemoresistance in CRC, many strategies are being investigated. Here, we review the common and novel measures to combat the resistance, including drug repurposing (nonsteroidal anti‐inflammatory drugs, metformin, dichloroacetate, enalapril, ivermectin, bazedoxifene, melatonin, and S‐adenosylmethionine), gene therapy (ribozymes, RNAi, CRISPR/Cas9, epigenetic therapy, antisense oligonucleotides, and noncoding RNAs), protein inhibitor (EFGR inhibitor, S1PR2 inhibitor, and DNA methyltransferase inhibitor), natural herbal compounds (polyphenols, terpenoids, quinones, alkaloids, and sterols), new drug delivery system (nanocarriers, liposomes, exosomes, and hydrogels), and combination therapy. These common or novel strategies for the reversal of chemoresistance promise to improve the treatment of CRC.

## Graphical abstract

Chemoresistance is the major cause of treatment failure and disease relapse in colorectal cancer (CRC). In addition to some common reversal measures, some novel strategies have sprouted in recent years, including drug repurposing, gene therapy, protein inhibitor, natural herbal compounds, new drug delivery system, and combination therapy, which promise to improve the treatment of CRC.

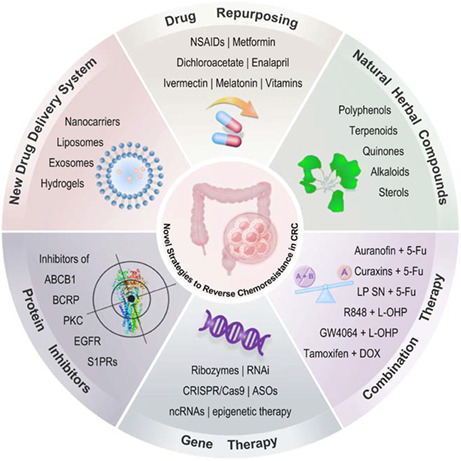

## 1 INTRODUCTION

Colorectal cancer (CRC) is the third most prevalent cancer around the world, with increasing morbidity and mortality every year.^1^ As reported by the International Agency for Research on Cancer (IARC) in 2020, there were over 193,000 new cases of CRC and more than 953,000 deaths worldwide, and CRC accounted for 10.0% of overall cancer incidence.^2^ Currently, the treatment of CRC mainly consists of surgery, targeted therapy, radiotherapy, and chemotherapy.^3^ Generally, chemotherapy, as the most classical and most common‐used therapy in CRC, can be applied at different stages of the treatment and is commonly provided after surgery as adjuvant therapy for patients with advanced CRC.^4^ Treatment with chemotherapeutic agents like 5‐fluorouracil (5‐FU), oxaliplatin (L‐OHP), vincristine (VCR), doxorubicin (DOX), cisplatin (CDDP)and irinotecan (CPT‐11) has improved the overall survival of patients with advanced CRC in the past decades.^5^ However, even though chemotherapy is well demonstrated to reduce tumor burden and prolong survival, it remains a palliative treatment since most CRC patients eventually exhibit drug resistance.^6^ More than 90% of patients with metastatic cancer may suffer chemotherapy failure due to drug resistance.^7^

**FIGURE 1:**
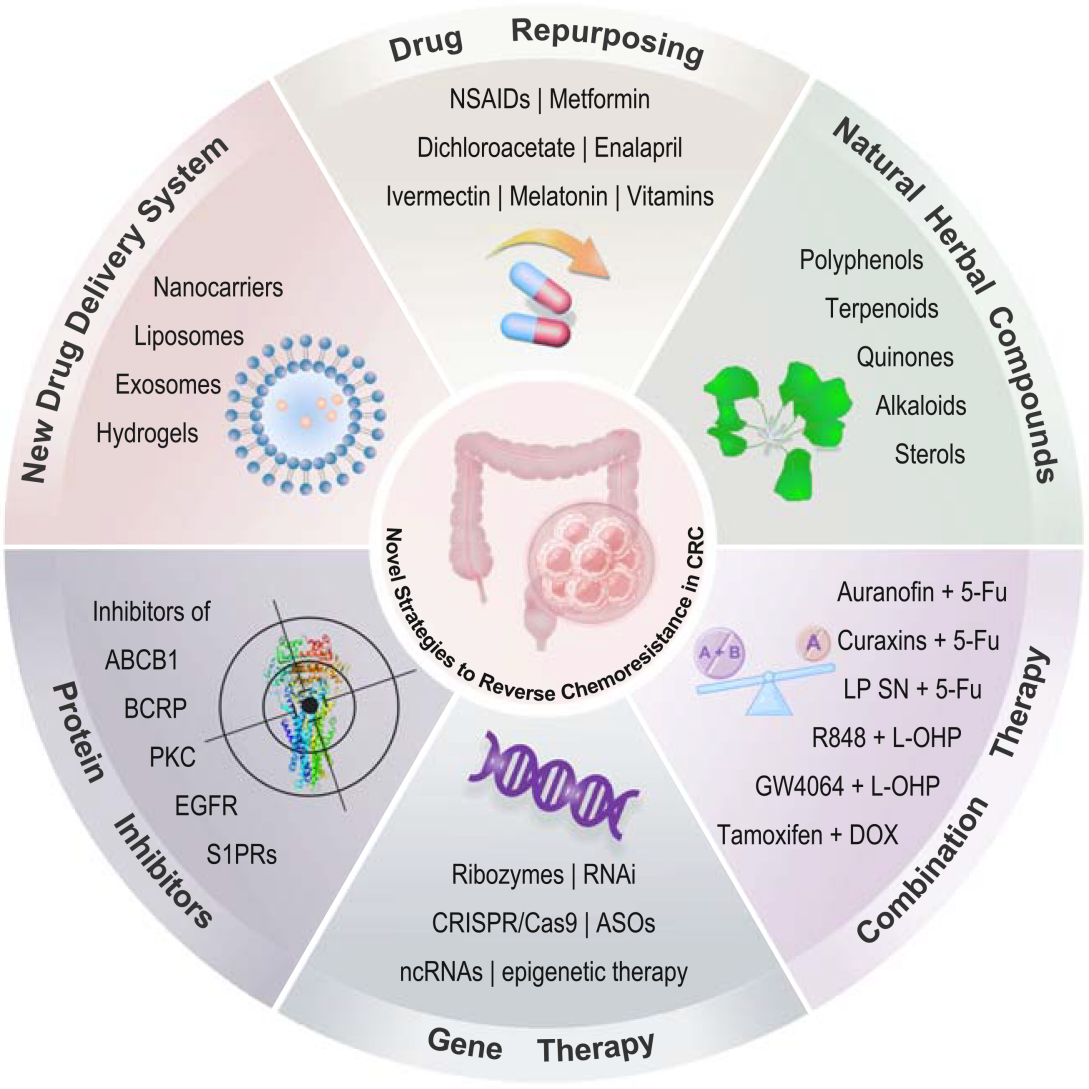
Novel strategies to reverse chemoresistance in colorectal cancer. ABCB1, ATP‐binding cassette subfamily B member 1; ASOs, antisense oligonucleotides; BCRP, breast cancer resistance protein; CRC, colorectal cancer; CRISPR/Cas9, clustered regularly interspaced short palindromic repeat/cas9; DOX, doxorubicin; EGFR, epidermal growth factor receptor; 5‐FU, 5‐fluorouracil; L‐OHP, oxaliplatin; LP SN, lactobacillus plantarum supernatant; ncRNAs, noncoding RNAs; NSAIDs, nonsteroidal anti‐inflammatory drugs; PKC, protein kinase C; RNAi, RNA interference; S1PRs, sphingosine 1‐phosphate receptors.

Chemotherapy resistance, according to drug responsiveness, can be classified into two types: intrinsic resistance and acquired resistance. It can also be divided into primary drug resistance and multidrug resistance (MDR) in terms of the drug resistance spectrum. Multidrug resistance is seem to be the major determinant of cancer chemotherapy failure. Nowadays, drug resistance not only undermines the therapeutic effects of anticancer chemical drugs but also causes CRC refractory. Therefore, drug resistance has become a thorny issue and urgently needed to be solved. Various strategies have been applied or investigated to the drug resistance reversal in CRC. The most common treatment among them is the combined use of chemical drugs and marketed medicines with already known effects such as drug repurposing (nonsteroidal anti‐inflammatory drugs, metformin, dichloroacetate, enalapril, ivermectin, bazedoxifene, melatonin, and S‐adenosylmethionine) and natural herbal compounds (polyphenols, terpenoids, quinones, alkaloids, and sterols). Gene therapy, as an emerging technology, has also been studied in chemoresistance reversal, including ribozymes, RNA interference, CRISPR/Cas9, epigenetic therapy, antisense oligonucleotides, and noncoding RNAs, which opens the door to a new genetical world in this field. Protein inhibitors are countermeasures at protein levels, which exserts their reversal effects directly by reacting with specific proteins such as EGFR inhibitor, S1PR2 inhibitor, and DNA methyltransferase inhibitor. It has been known that conventional drug delivery systems have many shortcomings comprising low bioavailability and cytotoxicity, which contribute to drug resistance. New drug delivery systems, including nanocarriers, liposomes, exosomes, and hydrogels, can overcome the drawbacks of traditional delivery systems and transport the therapeutic molecules directly to the specific tumor site, which plays an important role in reversing chemoresistance (Figure 1).

In this review, the aforementioned novel strategies to overcome chemoresistance in CRC will be clarified, which will provide new ideas for the treatment of drug resistance in CRC.

## 2 DRUG REPURPOSING

Recently, increased classical drugs, which were widely used in the clinic for different diseases, have been found to possess the ability to reverse chemoresistance in CRC, such as some nonsteroidal anti‐inflammatory drugs (aspirin, ibuprofen, and NS‐398), metformin, dichloroacetate, enalapril, ivermectin, bazedoxifene, melatonin, and S‐adenosylmethionine. Due to their low expenses, low toxicity, and high safety, drug repurposing is a promising strategy for combating chemoresistance in CRC (Table 1).

**TABLE 1:** Drug repurposing in the reversal of chemoresistance in CRC

Drug	Common uses	Chemical drugs	Cell model	Animal species	Animal model	Resistant mechanisms	Reference
Aspirin	Nonsteroidal anti‐inflammatory drugs: antipyretic, analgesic, and anti‐inflammatory	5‐FU	SW480/5‐FU, SW620/5‐FU cell lines	BALB/c nude mice (female, 6–8 weeks)	Xenograft	⬇5‐FU‐induced NF‐κB activation	^9^
		VCR	CAF‐like cells obtained by TGF‐β2 stimulation of HMEC‐1	—	—	⬇TGF‐βs and IL‐6	^10^
Ibuprofen		VCR	CAF‐like cells obtained by TGF‐β2 stimulation of HMEC‐1	**—**	**—**	⬇TGF‐βs and IL‐6	^10^
NS‐398		VCR	HCT‐8/VCR cell line	**—**	**—**	⬇MDR1, P‐gp and p‐c‐Jun	^11^
Metformin	The first‐line treatment for type 2 diabetes mellitus, suppresses hepatic glucose production	CDDP	SW480 and SW620 cell lines	**—**	**—**	⬇ROS production, PI3K/Akt signaling pathway	^14^
		CPT‐11	HCT1116 and SW480 cell lines	**—**	**—**	Block cell cycle in G1 and S phases	^15^
		5‐FU	SNU‐C5/5‐FU cell line	**—**	**—**	⬇DNA replication and NF‐κB ⬆AMPK	^16^
		5‐FU	HCT116 cell line	**—**	**—**	⬇stemness, EMT and Wnt3a/β‑catenin pathway	^17^
Combined use of metformin and propranolol	Propranolol (mainly used for hypertension)	5‐FU	HCT116/ 5‐FU cell line	BALB/c mice and athymic nude mice (female, 8 weeks)	Xenograft (HCT116 cells, HCT116/5‐FU cells); AOM/DSS model	⬇EMT	^18^
Dichloroacetate	Metabolic diseases	5‐FU	HCT‐8/5‐FU cell line	Nude mice (male, 6 weeks)	Xenograft (HCT‐8/5‐FU cells)	⬇p53/miR‐149‐3p/PDK2 glucose metabolic pathway	^19^
		L‐OHP	HCT‐116/L‐OHP cell line	BALB/c nude mice (male, 8 weeks)	Xenograft (HCT‐116/L‐OHP cells, HCT‐116/miR‐107‐overexpressing cells)	⬆AMPK‐mTOR pathway	^20^
Enalapril	Antihypertensive and anti‐heart failure	5‐FU	SW620 and HCT116 cell line	BALB/c nude mice (female, 6–8 weeks)	Xenograft (SW620 cells)	⬇NF‐κB/STAT3‐regulated proteins (c‐Myc, Cyclin D1, MMP‐9, MMP‐2, VEGF, Bcl‐2, and XIAP)	^21^
Ivermectin	Antiparasitic drug	VCR	HCT‐8/VCR cell line	BALB/c nude mice (female, 4‐week‐old)	Xenograft (VCR‐sensitive or resistant HCT‐8 cells)	⬇P‐gp, EGFR, ERK/Akt/NF‐κB signal	^22^
Bazedoxifene	Third‐generation selective estrogen receptor modulator and novel IL‐6/GP130 target inhibitor	5‐FU	HT29, SW480, LOVO and HCT116 cell lines	Athymic nude mice (female, 4–6 weeks)	Xenograft (HT29 cells)	⬇IL‐6/GP130 signaling pathway and phosphorylation of AKT, ERK and STAT3	^23^
Melatonin	Insomnia	5‐FU	SW480/5‐FU and HCT116/5‐FU cell lines	—	—	⬆miR‐215‐5p and ⬇TYMS	^24^
S‐Adenosylmethionine	Nutritional supplement	5‐FU	HCT 116^p53+/+^ and LoVo cell lines	—	—	⬇P‐gp and activation of NF‐κB	^25^
Vitamin C	Dietary supplements	L‐OHP	C2BBe1 and WiDr cell lines	**—**	**—**	⬇BAX/BCL2 ratio	^26^
Vitamin D		5‐FU	CBS, Moser, Caco‐2 and HCT116 cell lines	**—**	**—**	⬇TS and surviving in aCaSR‐dependent manner	^27^
1α,25‐dihydroxyvitamin D3		5‐FU/CPT‐11	5‐FU/CPT‐11‐resistant MIP101 human CRC cell line	**—**	**—**	⬆vitamin D receptor (VDR)	^28^

Note: “⬆” means upregulation or activation; “⬇” means downregulation or inhibition.

Abbreviations: AKT, protein kinase B; AMPK, AMP‐activated protein kinase; AOM, azoxymethane; CAFs, cancer‐associated fibroblast; CaSR, calcium‐sensing receptor; CDDP, cisplatin; CPT‐11, irinotecan; CPT‐11, irinotecan; DSS, dextran sodium sulfate; EGFR, epidermal growth factor receptor; EMT, epithelial mesenchymal transformation; ERK, extracellular regulated protein kinases; 5‐FU, 5‐fluorouracil; GP130, glycoprotein 130; HMEC‐1, human microvascular endothelial cells; IL‐6, interleukin‐6; L‐OHP, oxaliplatin; PDK2, MDR1, multidrug resistance protein 1; MMP‐2, matrix metalloproteinase‐2; MMP‐9, matrix metalloproteinase‐9; mTOR, mammalian target of rapamycin; NF‐κB, nuclear factor‐kappa B; p‐c‐Jun, phospho‐c‐Jun; P‐gp, P‐glycoprotein; PI3K, phosphatidylinositol 3 kinase; pyruvate dehydrogenase kinase 2; ROS, reactive oxygen species; STAT3, signal transducer and activator of transcription 3; TGF‐β, tumor growth factor‐β; TS, thymidylate synthase; TYMS, thymidylate synthase; VEGF, vascular endothelial growth factor; VCR, vincristine; VDR (vitamin D receptor); XIAP, X‐linked inhibitor of apoptosis protein.

### 2.1 Nonsteroidal anti‐inflammatory drugs

Nonsteroidal anti‐inflammatory drugs (NSAIDs), as a kind of widely used medicine worldwide, have potent anti‐inflammatory, analgesic, and antipyretic activity. The main mechanism of the action is the inhibition of cyclo‐oxygenase (COX), which is responsible for the biosynthesis of prostaglandins and thromboxane.^8^ Aspirin, ibuprofen, and NS‐398 are found to own the potential for overcoming drug resistance in CRC. It was reported that the combined use of aspirin (a nonselective COX inhibitor) and 5‐FU could reverse drug resistance and potentiate the antineoplasm effect of 5‐FU by abolishing 5‐FU‐induced nuclear factor‐kappaB (NF‐κB) activation in 5‐FU‐resistant SW480, SW620 (SW480/5‐FU and SW620/5‐FU) cell lines and xenograft mice of CRC.^9^ Wawro et al. found that both aspirin and ibuprofen inhibit the VCR‐dependent secretion of tumor growth factor‐βs (TGF‐βs) and interleukin‐6 (IL‐6) from cancer‐associated fibroblasts (CAFs) in CAF‐like cells, suggesting that aspirin and ibuprofen may reverse VCR‐resistance in CRC to some extent.^10^ NS‐398, a selective COX‐2 inhibitor, could significantly inhibit the expression of multidrug resistance protein 1 (MDR1) mRNA, P‐glycoprotein (P‐gp/ABCB1), phosphorylated‐c‐Jun levels and increase the intracellular concentration of VCR in VCR‐resistant HCT‐8/VCR cell line, promoting the sensitivity to VCR in resistant cells.^11^

### 2.2 Metformin

Metformin (MET), which suppresses hepatic glucose production, is the first‐line therapy for type 2 diabetes mellitus.^12^ MET has been recently demonstrated to reverse the resistance after chemotherapy and possess a potential therapeutic effect on colorectal cancer.^13^ MET enhanced the chemosensitivity of SW480 and SW620 cell lines to CDDP, inhibited cell proliferation, induced cell apoptosis, and increased the production of reactive oxygen species (ROS) through ROS‐mediated phosphatidylinositol 3 kinase (PI3K)/protein kinase B (AKT) signaling pathway.^14^ MET was also found to sensitize HCT1116 and SW480 cell lines to CPT‐11‐induced cytotoxicity and block the cell cycle in G1 and S phases, which indicates an innovative strategy in the treatment of CPT‐11‐resistant CRC.^15^ Moreover, MET could inhibit cell proliferation, migration, and cancer stem cell (CSC) population in 5‐FU‐resistant SNU‐C5 CRC cell line, which was mediated by the activation of AMP‐activated protein kinase (AMPK) and suppression of DNA replication, and NF‐κB pathway.^16^ MET was also found to resensitize HCT116 cells to 5‐FU resistance, which was realized by attenuating stemness and epithelial–mesenchymal transformation (EMT) via inhibiting the Wnt3a/β‐catenin pathway.^17^ Furthermore, it was found that the combined use of MET and propranolol (mainly used for hypertension treatment) could block 5‐FU chemoresistance in HCT116 cells to some extent, which could be applied as a putative adjuvant treatment for CRC and chemo‐resistant CRC.^18^

### 2.3 Dichloroacetate

Dichloroacetate (DCA), originally utilized to treat metabolic diseases as a metabolic regulator, was discovered to overcome chemoresistance in CRC through p53/miR‐149‐3p/pyruvate dehydrogenase kinase 2 (PDK2) glucose metabolic pathway.^19^ Liang et al. found that dichloroacetate could increase L‐OHP chemosensitivity in CRC by upregulating calcium‐binding protein 39 expression and then activating the AMPK‐mammalian target of the rapamycin (mTOR) pathway.^20^

### 2.4 Enalapril

Enalapril, an antihypertensive and antiheart failure drug, was found to inhibit cell migration and invasion and overcome chemoresistance in the 5‐FU‐resistant CRC cells at clinically acceptable dosages without extra toxicity. And the effect may be primarily achieved by the suppression of cell proliferation, angiogenesis, and NF‐κB/signal transducer and activator of transcription 3 (STAT3)‐regulated proteins, including Cyclin D1, c‐Myc, matrix metalloproteinase‐2 (MMP‐2), matrix metalloproteinase‐9 (MMP‐9), vascular endothelial growth factor (VEGF), Bcl‐2, and X‐linked inhibitor of apoptosis protein (XIAP).^21^

### 2.5 Ivermectin

Ivermectin, an antiparasitic drug, could reverse the chemoresistance in VCR‐resistant HCT‐8/VCR cells by diminishing P‐gp expression via reducing the activation of epidermal growth factor receptor (EGFR) and its downstream extracellular signal‐regulated kinases (ERK)/Akt/NF‐κB signaling pathway.^22^

### 2.6 Bazedoxifene

Bazedoxifene, the third‐generation of selective estrogen receptor modulator, also a novel IL‐6/glycoprotein 130 (GP130) target inhibitor, improved the anticancer effect of 5‐FU in CRC via impeding the IL‐6/GP130 signaling pathway and the phosphorylation of the downstream effectors such as AKT, ERK, and STAT3, suggesting that the block of IL‐6/GP130 might reverse chemoresistance in CRC.^23^

### 2.7 Melatonin

Melatonin, used for insomnia in usual, was found to reduce cell viability, promote apoptosis, and reverse 5‐FU resistance in SW480/5‐FU and HCT116/5‐FU cells by downregulating thymidylate synthase (TYMS) via upregulating miR‐215‐5p.^24^

### 2.8 S‐adenosylmethionine

S‐adenosylmethionine (AdoMet), a natural chemical and generally utilized as a dietary supplement, was capable of conquering 5‐FU chemoresistance in CRC cells through reverting the P‐gp upregulation induced by 5‐FU and suppressing the activation of the key anti‐apoptotic factor NF‐κB implicated in P‐gp‐related chemoresistance.^25^

### 2.9 Vitamins

Vitamins are common dietary supplement used in daily life and their potential effects on chemoresistance reversal in CRC have been found, including vitamin C and vitamin D. Vitamin C, also called ascorbic acid, could sensitize the human CRC cells to L‐OHP by inducing cell apoptosis.^26^ Vitamin D, which promotes calcium absorption and maintains bones healthy, could increase the sensitivity of human CRC cells to 5‐FU by suppressing TYMS and survivin expression in a calcium‐sensing receptor (CaSR) ‐dependent manner.^27^ Taghizadeh et al. found that the active metabolite of vitamin D 1α,25‐dihydroxy vitamin D3 (1,25‐D3) could restore the sensitivity to 5‐FU and CPT‐11 in 5‐FU/CPT‐11‐resistant MIP101 human CRC cell line. Furthermore, they also found that combined 1α,25‐dihydroxy vitamin D3 with secreted protein acidic and rich in cysteine (SPARC) which is a matricellular protein could augment chemosensitivity in CRC through upregulating the expression of vitamin D receptor (VDR) with a lower dosage of chemo drugs.^28^

## 3 GENE THERAPY

Gene therapy has become a novel strategy to conquer chemoresistance in CRC by gene correction, immunogene therapy, prodrug activation, oncolytic viruses, and other technologies manipulated on genes.^29^ Recently, various studies have confirmed the reversal effect of ribozymes, antisense oligonucleotides (ASOs), RNA interference (RNAi), clustered regularly interspaced short palindromic repeat (CRISPR)/Cas9, epigenetic therapy, and noncoding RNAs (ncRNAs), including micro‐RNAs (miRNAs), long noncoding RNAs (lncRNAs), and circular RNAs (circRNAs) in CRC (Table 2).

**TABLE 2:** Gene therapy investigated in the reversal of chemoresistance in CRC (2016~2022)

Gene therapy	Chemical drugs	Cell model	Animal species	Animal model	Reversal mechanisms	Reference
Ribozymes						
Anti‐MDR1 Rz	DOX	SW1116R/MDR cell line	BALB/c‐nu/nu nude mice (male, 6 weeks)	Xenograft (SW1116R MDR cells)	⬇p‐gp	^30^
Anti‐MRP Rz and Anti‐MDR1 Rz	DOX and VP‐16	HCT‐8/ CDDP cell line	—	—	⬇MRP and MDR1	^31^
Anti‐γ‐GCS Rz	CDDP, DOX and VP‐16	HCT‐8/ CDDP cell line	—	—	⬇γ‐GCS gene and ⬇MRP/MDR1	^32^
RNAi						
ADAM17 shRNA	L‐OHP	HCT‐8/L‐OHP cell line	—	—	⬇EGFR/PI3K/AKT signaling pathway	^41^
PIK3CA shRNA and PIK3CB shRNA	5‐FU	HCT‐8/5‐FU cell line	BALB/c nude mice (female, 6 weeks)	Xenograft (HCT‐8/5‐FU cells stably transfected with PIK3CA shRNA, PIK3CB shRNA)	⬇PIK3CA, PIK3CB, and MDR‐1	^42^
GOLPH3 shRNA	L‐OHP	HCT116/L‐OHP cell line	BALB/cSlc‐nu/nu mice (male, weighing 19 g ± 2 g)	Xenograft (HCT116/L‐OHP cells transfected with GOLPH3 shRNA)	⬇PI3K/AKT/mTOR pathway	^43^
Ufd1 siRNA	HCPT	SW1116/HCPT cell line	—	—	⬆caspase‐3 pathway and ⬇endoplasmic reticulum functions	^36^
CD147 shRNA	CDDP, DOX, GEM	HT29 cell line	—	—	⬇CD147	^44^
livin shRNA	VCR, VP‐16, 5‐FU	HCT‐8/VCR cell line	—	—	⬇livin (antiapoptotic)	^45^
KLK11 RNAi	L‐OHP	HCT‐8/L‐OHP cell line	—	—	⬆apoptosis and ⬇PI3K/AKT pathway	^40^
CES2 shRNA	L‐OHP	HCT116/L‐OHP and RKO/ L‐OHP cell lines	—	—	⬆apoptosis and ⬇PI3K/AKT pathway	^46^
SH3GL1 siRNA	5‐FU	LoVo/5‐FU, HT29/5‐FU, HCT8/5‐FU, and HCT116/5‐FU cell lines	—	—	⬇MDR1/P‐gp and EGFR/ERK/AP‐1 pathway	^37^
MDR1 siRNA	L‐OHP	SW480/L‐OHP cell line	—	—	⬇MDR1/P‐gp	^38^
FKBP3 shRNA	L‐OHP	primary CRC cells	—	—	⬇HDAC2, p‑AKT, ⬆PTEN and cleaved caspase‑3	^47^
CAC1 shRNA	5‐FU	SW480/5‐FU and LoVo/5‐FU cell lines	BALB/C, Nu/Nu nude mice (4‐6 weeks)	Xenograft (SW480/5‐FU cells transfected with CAC1 shRNA); tumor liver metastasis nude mice model	⬆apoptosis, ⬇MDR1/P‐gp	^48^
β3GnT8 siRNA	5‐FU	SW620 /5‐FU cell line	—	—	⬇polylactosamine formation	^39^
CD133 knockdown plasmid	DOX	DOX‐resistant LoVo/ADR and HCT8/ADR cell line	Athymic nude mice (male, 18–22 g)	Xenograft (LoVo/ADR^CD133 KD^ cells)	⬇CD133, AKT/NF‐κB/MDR1 pathway	^49^
CRISPR/Cas9						
ABCB1 knockout	DOX	MDR HCT8/VCR cell line	—	—	⬇ABCB1	^52^
ABCB1 knockout	PTX	SW620/Ad300 cell line	—	—	⬇ABCB1	^53^
RBX2 knockout		HCT116 and SW480 cell lines	BALB/c‐nude mice (5 weeks)	Xenograft (HCT116 or SW480 cells transfected with RBX2‐shRNA)	⬇mTOR/S6K1	^54^
TIAM1 knockdown	5‐FU	—	athymic nude mice (male, 5 weeks)	Xenograft (HCT116 cells with stably knocked down TIAM1)	⬇TIAM1	^55^
ASOs						
miR‐21i	5‐FU	HCT‐116/5‐FU cell line	—	—	⬇miR‐21, ⬆cell cycle arrest and apoptosis	^34^
Anti‐miR‐19a	L‐OHP	SW480/L‐OHP and HT29/L‐OHP cell lines	—	—	⬇ miR‐19a	^35^
MiRNAs						
miR‐200b‐3p	L‐OHP	HT29/L‐OHP and HCT116/L‐OHP cell lines	—	—	⬆miR‐200b‐3p, apoptosis ⬇βIII‐tubulin protein	^60^
miR‐506	L‐OHP	HCT116/L‐OHP cell line	—	—	⬆miR‐506, ⬇P‐gp, Wnt/β‐catenin pathway	^61^
miR‐27b‐3p	L‐OHP	SW480/L‐OHP cell line	—	—	⬆miR‐27b‐3p, ⬇autophagy, ATG10	^62^
miR‐195‐5p	5‐FU	HCT116/5‐FU and SW480/5‐FU cell line	—	—	⬆MiR‐195‐5p, ⬇GDPD5	^63^
miR‐133b	5‐FU, L‐OHP	5‐FU and L‐OHP chemoresistance in HT29 and SW620 cells	—	—	⬆MiR‐133b, ⬇DOT1L, stemness	^64^
miR‐375‐3p	5‐FU	HT29 and HCT116 cell lines	BALB/c nude mice (4 weeks)	Xenograft (HCT116 cells)	⬆MiR‐375‐3p, ⬇apoptosis and cell cycle arrest, TYMS	^65^
miR‐1914* and miR‐1915	5‐FU, L‐OHP	5‐FU and L‐OHP resistant HCT116 cells	—	—	⬆miR‐1914* and miR‐1915; ⬇NFIX	^66^
miR‐193a‐5p	5‐FU, L‐OHP	SW480, LS180, and HT‐29	—	—	⬆miR‐193a‐5p; ⬇CXCR4	^68^
miR‐195‐5p	5‐FU	5‐FU‐resistant SW620 and HT‐29 cell lines	—	—	⬆MiR‐195‐5p; ⬇Notch2 and RBPJ	^69^
miR‐135b and miR‐182	5‐FU	HCT‐8/5‐FU and LoVo/5‐FU cells			⬇miR‐135b, miR‐182, PI3K/AKT pathway; ⬆ST6GALNAC2	^70^
miR‐26b	5‐FU	HT‐29/5‐FU and LOVO/5‐FU cell lines	BALB/c‐nude mice (male, 4 weeks)	Xenograft (HT‐29/5‐FU/miR‐26b‐ cells)	⬆miR‐26b; ⬇p‐gp	^71^
LncRNAs						
lncRNA CCAT1	5‐FU	HCT116/5‐FU and HT29/5‐FU cells	—	—	⬇CCAT1	^73^
LINC00689	5‐FU	HCT116 and LoVo cell lines	BALB/c‐nude mice (male, 4‐5 weeks)	Xenograft (LINC00689+ and miR‐31‐5p‐ stable HCT116 and LoVo cells)	⬆LINC00689; ⬇miR‐31‐5p, YAP/β‐catenin pathway	^74^
MIR600HG	L‐OHP	Caco2 cell line	athymic (nu/nu) mice (female, 6 weeks)	Caco2 cells	⬆MIR600HG; ⬇stemness, ALDH1A3	^75^
lnc‐AP	L‐OHP	HCT116/L‐OHP and SW480/L‐OHP cell lines	—	—	encoding short peptide pep‐AP, which ⬇TALDO1, PPP; ⬆ROS, apoptosis	^76^
CircRNAs						
Circ_0032833	FOLFOX	HCT116/ FOLFOX cell line	—	—	⬇circ_0032833; ⬆MSI1/miR‐125‐5p	^78^
Circ‐PRKDC	5‐FU	SW480/5‐FU and SW620/5‐FU Cell lines	Nude mice (5 weeks)	Xenograft (SW480/5‐FU cells transfected with sh‐circ‐PRKDC)	⬇Circ‐PRKDC, wnt/β‐catenin pathway; ⬆FOXM1/miR‐375	^79^
circ_0007031	5‐FU	HCT116/5‐FU and SW480/5‐FU cell lines	BALB/c nude mice (6 weeks)	Xenograft (SW480/5‐FU cells transfected with sh‐circ‐0007031)	⬇circ_0007031; ⬆ABCC5/miR‐133b	^80^
circ_0094343	5‐FU, L‐OHP and Dox	HCT116 cell lines	—	—	⬆circ_0094343; ⬆TRIM67/miR‐766‐5p, glycolysis	^81^
circ_0006174	DOX	LoVo/DOX and HCT116/DOX cell lines	—	—	⬆Circ_0006174; ⬇CCND2/miR‐1205	^82^
circCSPP1	DOX	LoVo/DOX and HCT116/DOX cell lines	nude mice (6 weeks)	Xenograft (LoVo/DOX cells transfected withsh‐circCSPP1)	⬇circCSPP1, ⬆FZD7/miR‐944	^83^
circ_0071589	CDDP	HCT116/CDDP and LOVO/CDDP cell line	BALB/c nude mice (male, 5 weeks)	Xenograft (HCT116/CDDP cells transfected with sh‐circ_0071589)	⬇circ_0071589, KLF12; ⬆miR‐526b‐3p	^84^
CircEXOC6B	5‐FU	SW620 and HCT116 cell lines	—	—	competitively binding with RRAGB, ⬇HIF1A‐RRAGB‐mTORC1	^85^
Epigenetic therapy						
Zebularine (DNMT inhibitor)	L‐OHP	hypoxia‐induced oxaliplatin resistance in HCT116 cell line	BALB/c nude mice; C57BL/6 mice	Xenograft (HCT116 cells); AOM/DSS‐induced CRC mouse models	⬇HIF‐1α	^88^
Tunicamycin (N‐glycosylation inhibitor)	DOX	LoVo/DOX cell line	—	—	⬇P‐gp and BCRP	^92^
METTL1 (mediates m7G methylation)	CDDP	HCT116, SW480 and SW620	—	—	⬆METTL1, p53; ⬇miR‐149‐3p, S100A4	^91^

*Note*: “⬆” means upregulation or activation; “⬇” means downregulation or inhibition.

Abbreviations: ABCB1, ATP binding cassette subfamily B member 1; ABCC5, ATP‐binding cassette subfamily C member 5; ADAM17, A disintegrin and metalloproteinase 17; AKT, protein kinase B; ALDH1A3, aldehyde dehydrogenase 1 family, member A3; AOM, azoxymethane; AP‐1, activator protein‐1; ASOs, antisense oligonucleotides; anti‐miR‐19a, antisense oligonucleotide of miR‐19a; ATG10, autophagy‐related 10BCRP, breast cancer resistance protein; CAC1, CDK2‐associated cullin domain 1; CCAT1, colon cancer‐associated transcript 1; CCND2, cyclin D2; CDDP, cisplatin; CES‐2, carboxylesterase‐2; CircRNAs, circular RNAs; CSPP1, centrosome and spindle pole associated protein 1; CXCR4, C‐X‐C Motif chemokine receptor 4; DNMT, DNA methyltransferase; DOT1L, disruptor of telomeric silencing 1‐like; DOX, doxorubicin; EGFR, epidermal growth factor receptor; ERK, extracellular regulated protein kinases; DSS, dextran sodium sulfate; FKBPs, FK506‑binding proteins; FOLFOX, folinic acid, fluorouracil, and oxaliplatin; FOXM1, Forkhead box protein M1; 5‐FU, 5‐fluorouracil; FZD7, frizzled‐7; γ‐GCS, γ‐glutamylcysteine synthetase; GDPD5, glycerophosphodiester phosphodiesterase domain containing 5; GEM, gemcitabine; γ‐GCS, gamma‐glutamylcysteine synthetase; GOLPH3, golgi phosphoprotein 3; HCPT, hydroxycamptothecin; HDAC2, histone deacetylase 2; HIF‐1α, hypoxia inducible factor‐1α; KLK11, Kallikrein 11; KLF12, Krüppel‐like factor 12; L‐OHP, oxaliplatin; LncRNAs, long non‐coding RNAs; MDR, multidrug resistance; MDR1, multidrug resistance protein 1; METTL1, methyltransferase‐like 1; METTL3, methyltransferase‐like 3; m7G, 7‐methylguanosine; MiRNAs, micro RNAs; MRP, multidrug resistance‐associated protein; MSI1, musashi1; mTOR, mammalian target of rapamycin; NFIX, nuclear factor I/X; p‐AKT, phosphorylated AKT; P‐gp, P‐glycoprotein; PI3K, phosphatidylinositol 3 kinase; PPP, pentose phosphate pathway; PTEN, phosphatase and tensin homolog deleted on chromosome 10; PTX, paclitaxel; RBPJ, recombination signal binding protein for immunoglobulin Kappa J region; RBX2, RING box protein 2; RNAi, RNA interference; Rz, ribozyme; RRAGB, Ras‐related GTP binding protein B; SH3GL1, Src homology 3 (SH3)‐domain GRB2‐like protein 1; shRNA, short hairpin RNA; S6K1, S6 kinase 1; TALDO1, transaldolase 1; TIAM1, T‐lymphoma invasion and metastasis‐inducing protein‐1; TRIM67, tripartite motif‐containing 67; TYMS, thymidylate synthase; Ufd1, ubiquitin fusion‐degradation 1‐like protein; VP‐16, etoposide; Yap, yes‐associated protein.

### 3.1 Ribozymes

Ribozymes are small RNA molecules with catalytic functions, which can degrade specific mRNA sequences. Transfection of the anti‐MDR1 ribozyme, bound to the carcino‐embryonic‐antigen (CEA) promoter, could reverse DOX resistance by reducing P‐gp expression in SW1116R MDR CRC cells.^30^ Hammerhead ribozyme‐mediated‐specific suppression of MDR1 and multidrug resistance‐associated protein (MRP) could reverse resistance to DOX and etoposide (VP‐16) in CDDP‐resistant HCT‐8/CDDP cells.^31^ Hammerhead ribozyme against γ‐glutamylcysteine synthetase (γ‐GCS) could reverse resistance to CDDP, DOX, and VP‐16 in CDDP‐resistant HCT‐8 cells by markedly downregulating the expression of the γ‐GCS gene and MRP/MDR1.^32^

### 3.2 Antisense oligonucleotides

Antisense oligonucleotides (ASOs) are synthesized nucleic acids, containing 12–25 nucleotides in general, which exert their inhibitive effect by binding to the target RNA through Watson–Crick hybridization.^33^ It was reported that combined use of miR‐21 inhibitor oligonucleotide (miR‐21i) and 5‐FU delivered by the engineered exosomes could overcome resistance and markedly enhance the drug cytotoxicity in 5‐FU‐resistant HCT‐116/5‐FU cell line by increasing apoptosis, inducing cell cycle arrest, reducing tumor proliferation, and rescuing the decrease of phosphatase and tensin homolog (PTEN) and hMSH2 (regulatory targets of miR‐21) due to the downregulation of miR‐21.^34^ The antisense oligonucleotide of miR‐19a (anti‐miR‐19a) has been found to reverse L‐OHP resistance in L‐OHP‐resistant SW480 and HT29 cell lines via PTEN /PI3K/AKT pathway.^35^

### 3.3 RNA interference

RNA interference (RNAi) regulates gene expression and contributes to the targeted therapy through 20–24 bp RNA including small interfering RNAs (siRNAs) and short hairpin RNAs (shRNAs).

#### 3.3.1 Small interfering RNAs

Knockdown of the ubiquitin fusion‐degradation 1‐like protein (UFD1) could restore the sensitivity of hydroxycamptothecin (HCPT)‐resistant SW1116/HCPT colon cancer cell line to HCPT probably via increasing caspase‐3‐mediated apoptosis and inducing endoplasmic reticulum (ER) stress.^36^ Suppression of Src homology 3‐domain GRB2‐like protein 1 (SH3GL1) could reverse the 5‐FU resistance in a multitude of CRC cell lines including HT29/5‐FU, HCT116/5‐FU, LoVo/5‐FU, and HCT8/5‐FU cell lines through downregulating MDR1/P‐gp via the EGFR/ERK/activator protein‐1 (AP‐1) pathway.^37^ Silencing MDR1 could reverse the drug resistance in SW480L/OHP cells by downregulating MDR1 gene expression and reducing the gp level.^38^ Knockdown of β‐1,3‐N‐acetyl glucosaminyltransferase 8 (β3GnT8), which synthesizes polylactosamines on β1‐6 branched N‐glycans and with high expression in 5‐FU‐resistant SW620/5‐FU cell line, could reverse 5‐FU resistance via the suppression of the polylactosamines formation.^39^ RNAi of kallikrein 11 (KLK11) that is correlated with malignant behaviors of CRC can overcome L‐OHP resistance by inducing apoptosis and evading cell growth by suppressing the PI3K/AKT signaling pathway in L‐OHP‐resistant HCT‐8/L‐OHP cell line.^40^

#### 3.3.2 Short hairpin RNAs

A disintegrin and metalloproteinase 17 (ADAM17) shRNA could inhibit cell proliferation, increase apoptosis, and reverse L‐OHP resistance in L‐OHP resistant HCT‐8 cell line by suppressing the EGFR/PI3K/AKT signaling pathway.^41^ Silencing PIK3CA and PIK3CB by RNAi could repress the capability of proliferation, migration, and invasion of CRC cells and reverse MDR in 5‐FU‐resistant HCT‐8/5‐FU cell line.^42^ GOLPH3 shRNA could reverse L‐OHP resistance by limiting cell proliferation and inducing apoptosis through the suppression of P13K/AKT/mTOR pathway in L‐OHP resistance HCT116/L‐OHP cell line.^43^ RNAi of CD147 was observed to inhibit cell proliferation and invasion and resensitize HT29 cells to CDDP and DOX.^44^ Transfected of livin (an inhibitor of apoptosis proteins) shRNA to VCR resistant HCT‐8/VCR cells could increase apoptosis in response to VCR, VP‐16, and 5‐FU, which is able to reverse drug resistance.^45^ RNAi of carboxylesterase‐2 can suppress proliferation and induce apoptosis and reverse L‐OHP resistance by suppression of PI3K signaling in L‐OHP‐resistant HCT116/L‐OHP and RKO/L‐OHP cell lines.^46^ Overexpression of nuclear protein FK506‐binding protein 3 (FKBP3) and histone deacetylase 2 (HDAC2) could promote L‐OHP resistance in CRC cells, whereas knockdown of FKBP3 could abate L‐OHP resistance by decreasing HDAC2 expression and perhaps through regulating PTEN/AKT pathway in CRC cells.^47^ Knockdown of CDK2‐associated cullin domain 1 (CAC1), an innovative regulator of cell cycle, could promote the sensitivity of SW480/5‐FU and LoVo/5‐FU cell lines to 5‐FU by inducing cell apoptosis, arresting tumor cells at the G1/S phase, and lowering the expression of P‐gp and MRP‐1.^48^ Knockdown of CSC biomarker CD133 could reverse DOX chemoresistance by downregulating MDR1/P‐gp expression via AKT/NF‐κB/MDR1 signaling in DOX‐resistant LoVo and HCT8 cell lines.^49^ Knockout of SLC25A22 increased chemosensitivity of KRAS‐mutant DLD1 and SW1116 cell lines to 5‐FU, and it is also affirmed in DLD1 xenograft mice.^50^

### 3.4 CRISPR/Cas9

CRISPR/Cas9 technology, a powerful, efficient, easy, and specific gene editing tool, is extensively administrated in tumor pathogenesis, treatment, and drug resistance by inserting or knocking out the related genes.^51^ Identifying and modulating resistance‐related genes by CRISPR/Cas9 is an efficient way to reverse chemoresistance and inhibit tumor recurrence. Knockout ABCB1 gene by CRISPR/Cas9 tool increased the chemosensitivity of ABCB1 overexpressed MDR HCT8/VCR cell line to DOX by increasing intracellular accumulation of DOX.^52^ Similarly, Lei et al. also found that ABCB1 knockout could reverse 3H‐paclitaxel (PTX) resistance mediated by ABCB1 in SW620/Ad300 CRC cells by decreasing drug efflux.^53^ Knockout of redox‐inducible antioxidant protein RING‐box 2 (RBX2) by CRISPR/Cas9 technique enhanced the sensitivity of HCT116 and SW480 cell lines to PTX probably by deactivating mTOR/S6 kinase 1 (S6K1).^54^ Knockdown of T‐lymphoma invasion and metastasis‐inducing protein‐1 (TIAM1) enhanced sensitivity to 5‐FU and reduced tumor invasiveness in HCT116 xenograft mice.^55^ Apart from gene knockout, CRISPR/Cas9 can screen resistance‐associated genes by whole‐genome screening. Lan et al. found that TRAF5, a critical mediator of necroptosis, confers to L‐OHP‐resistance METTL3‐deleted HCT‐116 cells.^56^

### 3.5 Micro‐RNAs

Micro‐RNAs (MiRNAs), defined as small ncRNAs containing 21–23 nucleotides, regulate gene expression at the level of posttranscription by repressing mRNA translation and inducing mRNA degradation.^57^ Dysregulation of miRNAs is associated with colorectal carcinogenesis, and appropriate modulation of miRNAs is found to exert reversal effects in resistant CRC cells.^58^, ^59^ Wu et al. found that upregulation of miR‐200b‐3p could promote cell migration, induce apoptosis, inhibit cell growth, and reverse L‐OHP resistance by downregulating the expression of βIII‐tubulin protein through binding to 3′‐UTR of TUBB3 (encoding β III‐tubulin protein) in L‐OHP‐resistant HT29/L‐OHP and HCT116/L‐OHP cell lines.^60^Zhou et al. reported that overexpression of miR‐506 improved chemosensitivity in HCT116/L‐OHP cell line by suppressing P‐gp expression through downregulating Wnt/β‐catenin pathway.^61^ Sun et al. indicated that miR‐27b‐3p could reverse chemoresistance by inhibiting autophagy via inhibiting the expression of autophagy‐related protein ATG10 in L‐OHP‐resistant SW480 cells.^62^ MiR‐195‐5p could dramatically increase chemosensitivity in 5‐FU‐resistant HCT116/5‐FU and SW480/5‐FU CRC cell lines by reducing the expression of glycerophosphodiester phosphodiesterase domain containing 5 (GDPD5) that is involved in the process of choline phospholipid metabolism.^63^ MiR‐133b reduced stemness and 5‐FU and L‐OHP chemoresistance in HT29 and SW620 cell lines by targeting and suppressing the disruptor of telomeric silencing 1‐like (DOT1L)‐mediated H3K79me2 modification and transcription of stem cell genes.^64^ MiR‐375‐3p was discovered to be downregulated in CRC tissues as well as HT29 and HCT116 cell lines. It strengthened the sensitivity to 5‐FU in CRC cells by inducing cell cycle arrest and apoptosis via targeting TYMS.^65^ Upregulation of miR‐1914* and miR‐1915 decreased chemoresistance in 5‐FU and L‐OHP chemo‐resistant HCT116 cells by repressing the expression of transcription factor nuclear factor I/X (NFIX).^66^ MiR‐138‐5p, which decreased in CRC tissues, could inhibit cell migration and chemoresistance in CRC by targeting the nuclear factor I/B (NFIB)‐Snail1 axis.^67^ Combined with 5‐FU and L‐OHP, miR‐193a‐5p reversed chemoresistance in CRC by reducing C‐X‐C motif chemokine receptor 4 (CXCR4, induces cell proliferation and metastasis) expression.^68^ MiR‐195‐5p considerably increased tumor cell apoptosis, reduced tumor sphere formation, and prevented cell stemness and chemoresistance by decreasing Notch signaling proteins Notch2 and recombination signal binding protein for immunoglobulin Kappa J region (RBPJ).^69^ Suppression of miR‐135b and miR‐182 may reverse the 5‐FU resistance in HCT‐8/5‐FU and LoVo/5‐FU cell lines by targeting sialyltransferase ST6GALNAC2 via PI3K/AKT pathway.^70^ Overexpression of miR‐26b could reverse 5‐FU chemoresistance by downregulation of P‐gp in 5‐FU‐resistant HT‐2 and LOVO cell lines.^71^

### 3.6 Long noncoding RNAs

Long noncoding RNAs (LncRNAs), which are transcripts that exceed 200 nucleotides, can regulate CRC progression by acting as competitive endogenous RNAs (ceRNAs) by interacting with miRNAs or protein‐coding mRNAs and encoding short peptides.^72^ A growing number of studies has indicated that abnormally expressed lncRNAs confer to drug resistance in CRC and the condition can be overcome by re‐modulating the expression of the aberrant lncRNAs. Downregulation of lncRNA colon cancer‐associated transcript 1 (CCAT1) could effectively inhibit CCAT1 expression and reverse the 5‐FU resistance in HCT116/5‐FU and HT29/5‐FU cells.^73^ Long intergenic nonprotein‐coding RNA 689 (LINC00689) functioned as a miR‐31‐5p sponge to inhibit tumor proliferation, metastasis, and chemoresistance by upregulating large tumor suppressor kinase (LATS2) and repressing yes‐associated protein (YAP)/β‐catenin signaling pathway in CRC.^74^ LncRNA MIR600HG functioned as a tumor suppressor and the overexpression of MIR600HG could inhibit tumor invasion and enhance chemosensitivity by targeting ALDH1A3 (encodes an aldehyde dehydrogenase enzyme) in CRC.^75^ LncRNA lnc‐AP could sensitize the HCT116/L‐OHP and SW480/L‐OHP cell lines to L‐OHP by encoding short peptide pep‐AP, which suppressed pentose phosphate pathway (PPP), increased ROS accumulation and cell apoptosis through inhibiting transaldolase 1 (TALDO1, a key enzyme of PPP).^76^

### 3.7 Circular RNAs

CircRNAs, a special subclass of ncRNAs, have a covalently closed circular structure with no 5′ cap structure and 3′ polyA tail, which is associated with tumor recurrence and chemotherapy resistance in CRC.^77^ CircRNAs could participate in chemoresistance by acting as miRNAs sponges or binding with proteins. Circ_0032833 downregulated the expression of Musashi1 (MSI1), which promoted drug resistance in cancer by sponging miR‐125‐5p. Knockdown of circ_0032833 could sensitize folinic acid, fluorouracil, and oxaliplatin (FOLFOX)‐resistant HCT116 cells to 5‐FU and L‐OHP.^78^ Circ‐PRKDC knockdown suppressed 5‐FU Resistance in SW480/5‐FU and SW620/5‐FU Cell lines by directly targeting miR‐375, which negatively regulated the expression of transcription factor forkhead box protein M1 (FOXM1).^79^ Knockdown of circ_0007031 could repress tumor malignant progression and reverse 5‐FU resistance in HCT116/5‐FU and SW480/5‐FU cell lines via regulating ATP‐binding cassette subfamily C member 5 (ABCC5) expression by sponging miR‐133b.^80^ Circ_0094343 was found to inhibit tumor proliferation, clone formation, and glycolysis and improve the chemosensitivity to gemcitabine (GEM), 5‐FU, L‐OHP, and DOX in HCT116 cells via the miR‐766‐5p/ tripartite motif‐containing 67 (TRIM67) axis.^81^ Circ_0006174 was highly expressed in DOX‐resistant CRC tissues and cells. Downregulation of circ_0006174 could inhibit chemoresistance and tumor progression in DOX‐resistant CRC cells by upregulating the miR‐1205‐mediated cyclin D2 (CCND2).^82^ CircCSPP1 knockdown inhibited tumor growth and increased drug sensitivity of DOX‐resistant CRC cells by targeting miR‐944/frizzled‐7 (FZD7, a receptor of Wnt signaling proteins) axis.^83^ Circ_0071589 knockdown suppressed chemoresistance in CDDP‐resistant CRC cells via miR‐526b‐3p/Krüppel‐like factor 12 (KLF12) axis.^84^ CircEXOC6B could enhance the sensitivity of SW620 and HCT116 cell lines to 5‐FU by competitively binding with Ras‐related GTP binding protein B (RRAGB, activator of mTORC1 pathway), thus blocking the HIF1A‐RRAGB‐mTORC1 positive feedback loop.^85^

### 3.8 Epigenetic therapy

Epigenetic therapy is a coping strategy to modify the aberrant epigenetic alterations in cancers, including CRC. It has been found that epigenetic alterations like higher DNA methylation and mRNA N6‐methyladenosine (m6A) are related to the occurrence of chemoresistance and tumor relapse in CRC.^86^ Block the epigenetic modification that occurred in resistant CRC can reverse the resistance. Baharudin et al. found that recurrent CRC patients exhibited higher methylation levels and the recurrence of CRC compared to non‐recurrent CRC patients, which might associate with the abnormal methylation of *CCNEI*, *CCNDBP1*, *CHL1, DDX43*, and *PON3*. Treatment of 5‐aza‐2′‐deoxycytidine (5‐azadC, a DNA methylation inhibitor) could restore the sensitivity to 5‐FU in the SW48 cell line.^87^ Zebularine, a low‐toxicity DNA methyltransferase (DNMT) inhibitor, could overcome hypoxia‐induced CDDP resistance in HCT116 cells and show the same efficacy in HCT116 xenograft models and AOM/DSS‐induced CRC mouse models by downregulating HIF‐1α expression through hydroxylation.^88^ Zhang et al. found that methyltransferase‐like 3 (METTL3) and mRNA N6‐methyladenosine (m6A) were upregulated in 5‐FU‐resistant HCT‐116 and SW480 cell lines. Knockdown of METTL3 could restore the chemosensitivity of HCT‐116/5‐FU, SW480/5‐FU, and SW620/5‐FU cell lines mediated by downregulating the expression of lactate dehydrogenase (LDH) A, which catalyzes pyruvate to lactate to promote glycolysis.^89^ Uddin et al. found that silencing of METTL3 or inhibition of RNA methylation could restore the sensitivity to DOX in cells carrying the mutation of R273H by suppressing the m6A modification in the pre‐mRNA of p53 and increasing the phosphorylated level of p53 protein. In addition, suppression of ceramide glycosylation was also found to combat drug resistance by mechanisms of the suppression of METTL3 and m6A formation in p53 pre‐mRNA.^90^ Overexpression of methyltransferase‐like 1 (METTL1) was found to sensitize CDDP‐resistant colon cancer cells to chemoresistance via modulating miR‐149‐3p/S100A4/p53 axis.^91^ Wojtowicz et al. found that tunicamycin, an inhibitor of N‐glycosylation, might reverse MDR in LoVo/DOX cell line by blocking the first‐step N‐glycosylation and translocation of P‐gp.^92^

## 4 PROTEIN INHIBITORS

In the treatment of CRC chemoresistance, except for gene‐level therapy, direct protein inhibitors also play an important role. Various inhibitors, including inhibitors of classical resistance‐related proteins, EGFR inhibitors, and sphingosine 1‐phosphate receptor (S1PR) modulators have been found to reverse chemoresistance in CRC.

### 4.1 Inhibitors of classical resistance‐related proteins

An increment in drug efflux could reduce drug concentration and accumulation in tumor cells, which leads to drug resistance. Targeting ATP‐binding cassette (ABC) transporters like breast cancer resistance protein (BCRP) and P‐gp provides a potential approach to eliminating drug resistance during the treatment of CRC. GF120918, a potent P‐gp and BCRP inhibitor, markedly increased the oral bioavailability of topotecan from 40% to 97% in cancer patients.^93^ Compound WS‐10, a P‐gp inhibitor, could overcome P‐gp‐mediated MDR in SW620/Ad300 cells by binding with P‐gp, thus enhancing the intracellular accumulation of PTX.^94^ The protein kinase C (PKC) family, deemed as oncoprotein historically, is comprised of a group of serine or threonine kinases and is incorporated in tumor progression.^95^ It has been indicated that PKC could reduce intracellular drug concentration by activating ATP‐dependent efflux pumps, which contributes to drug resistance in CRC.^96^ Go6976, a specific inhibitor of classical PKC, can be used to reverse DOX resistance in human colon cancer DOX‐resistant HCT15 cell line by reducing MDR expression and increasing DOX‐induced apoptosis.^97^

### 4.2 EGFR Inhibitors

EGFR is a transmembrane receptor belonging to a family of receptor tyrosine kinases that mediates a series of intracellular pathways that promote tumor proliferation, invasion, metastasis, and neovascularization.^98^ EGFR inhibitors application has been found to overcome chemoresistance in CRC. Saptinib, an EFGR inhibitor, could increase 3H‐PTX accumulation in tumor cells and reverse MDR in SW720/Ad300 cells through stimulating ATPase which competitively inhibits 3H‐PTX uptake.^99^ Erlotinib, a specific inhibitor of EGFR, could effectively strengthen the antitumor effect of 5‐FU via modulating the EGFR‐FGD5‐AS1‐miR‐330‐3p‐hexokinase 2 (HK2) pathway.^100^

### 4.3 S1PR modulators

Sphingosine‐1‐phosphate (S1P), one of the sphingolipids, could regulate cancer survival by binding sphingosine 1‐phosphate receptors (S1PRs), which activate multiple cell growth‐related pathways.^101^ Inhibition of S1PR has been found to reverse chemoresistance in several cancers including CRC. JTE‐013, an S1P and S1PR2 inhibitor, could overcome 5‐FU resistance in CRC by lowering the expression of dihydropyrimidine dehydrogenase (DPD or DPYD).^102^ S1PR2 antagonists compound 40 could markedly reverse the drug resistance in HCT116/5‐FU and SW620/5‐FU cell lines by evading the expression of dihydropyrimidine dehydrogenase (DPD). It significantly enhanced the inhibitory effect of 5‐FU in the SW620/5‐FU cells xenograft murine model without significant liver toxicity.^103^

### 4.4 Others

Notch and Wnt signaling‐associated proteins were upregulated in HCT116/5‐FU and HCT116/L‐OHP cells, including Notch1 receptor intracellular domain NICD1 and nonphosphorylated β‐catenin and Notch target gene HES1. Wnt inhibitor XAV939 and Notch inhibitor RO4929097 could restore cell viability in L‐OHP‐treated HCT116 cells.^104^ Regorafenib, a multikinase inhibitor, which targets the RAS/RAF/MEK/ERK pathway, could overcome the ABCB1‐mediated MDR and increase 3H‐PTX accumulation in ABCB1‐overexpressing tumor cells by suppressing the efflux activity of ABCB1.^105^

## 5 NATURAL HERBAL COMPOUNDS

Natural herbal compounds have become putative adjuncts of conventional chemotherapy in CRC and are able to reverse acquired drug resistance by different mechanisms.^106^ Natural herbal compounds can be classified into polyphenols, terpenoids, quinones, alkaloids, sterols, and others according to the chemical structures (Table 3).

**TABLE 3:** Natural herbal medicine applied in the reversal of chemoresistance in CRC (2011–2022)

Chemical structure	Drug	Reversal Mechanisms	Resistant cell line	Reference
Polyphenols	Dihydromyricetin (flavonoid)	Inhibit MRP2 expression and its promoter activity by preventing NF‐κB‐Nrf2 signaling	HCT116/L‐OHP, HCT8/VCR	^103^
	Curcumin(non‐flavonoid)	Inhibition of proliferation, inducement of apoptosis, block of G0/G1 phase and expression of TET1 and NKD2; inhibit proliferation, increase apoptosis, downregulate P‐gp and HSP‐27	HCT‐116/5‐FU; HCT‐8/5‐FU	104, 105
	Resveratrol(non‐flavonoid)	Inhibiting EMT via up‐regulation of intercellular junctions and down‐regulation of NF‐κB pathway; downregulating MDR1, inhibiting NF‐kB pathway and the transcriptional activity of CRE	HCT116/5‐FU and SW480/5‐FU; HCT116/L‐OHP	106, 107
Terpenoids	β‐elemene (sesquiterpene)	Inhibit proliferation, induce pro‐death autophagy and Cyclin D3‐dependent cycle arrest	HCT116 (p53^−/−^)/5‐FU	^108^
	Atractylenolide II (sesquiterpenoid)	Inhibit cell proliferation and alleviate chemoresistance	Lovo, SW480/5‐FU, mitomycin, adriamycin and CDDP	^109^
Quinones	Emodin (anthraquinone derivative)	Inhibit proliferation, invasion, migration, and induce cell apoptosis and downregulate PI3K/Akt pathway	SW480/5‐FU	^110^
	Tanshinone IIA (diterpene quinone)	Decrease the levels of Bcl‐2, p‐Akt and p‐ERK, and increase the levels of Bax and active caspase 3 by inhibiting ERK/Akt Signaling Pathway	SW480/L‐OHP	^112^
	Cryptotanshinone	Inhibit tumor growth through induction of autophagic cell death and p53‐independent cytotoxicity	SW620 Ad300	^113^
	Dihydrotanshinone			
	Hypericin (anthraquinone)	Downregulation of MRP2 level, GSH‐related detoxification and NER‐mediated DNA repair mediated by ROS	HCT8/L‐OHP and HCT116/L‐OHP	^114^
Alkaloids	Evodiamine	Inhibit cell growth, induce apoptosis, suppress the expression of ABCG2 and inhibit p50/p65 NF‐κB Pathway	HCT‐116/L‐OHP	^115^
Sterols	Ginsenoside Rh2	Inhibit cell proliferation and migration, induce apoptosis, and decrease the expression of MRP1, MDR1, LRP and GST	LoVo/5‐FU, HCT‐8/5‐FU CRC	^116^
	β‐Sitosterol	Suppress BCRP, activate p53, and enhance apoptosis	HCT116/L‐OHP	^117^
Others	Salvianolic acid B (phenolic acid)	Increase ROS levels, promote apoptosis and downregulate the expression of P‐gp	HCT‑8/VCR	^118^

Abbreviations: ABCG2, ATP‐binding cassette superfamily G member 2; AKT, protein kinase B; BCRP, breast cancer resistance protein; CAFs, cancer‐associated fibroblasts; CDDP, cisplatin; CSCs, colorectal cancer stem cells; CRC, colorectal cancer; CRE, cAMP‐responsive element; DOX, doxorubicin; EGFR, epidermal growth factor receptor; ERK, extracellular regulated protein kinases; EMT, epithelial‐mesenchymal transition; 5‐FU, 5‐fluorouracil; GSH, glutathione; GST, glutathione S‐transferase; HCPT, hydroxycamptothecin; HIF‐1α, hypoxia‐inducible factor; Hsp27, heat shock protein 27; LncRNAs, long non‐coding RNAs; L‐OHP, oxaliplatin; LRP, lung resistance‐related protein; MDR1, multidrug resistance protein 1; miRNAs, micro RNAs; MRP1, multidrug resistance‐associated protein 1; MRP 2, multidrug resistance‐associated protein 2; ncRNAs, noncoding RNAs; NER, nucleotide excision repair; NF‐κB, nuclear factor‐kappa B; NKD2, naked cuticle homolog 2; Nrf2, nuclear factor E2‐related factor 2; NSAIDs, nonsteroidal anti‐inflammation drugs; p‐AKT, phosphorylated AKT; PCD, programmed cell death; p‐ERK, phosphorylated ERK; P‐gp, P‐glycoprotein; PI3K, phosphatidylinositol 3 kinase; PTEN, phosphate and tension homology deleted on chromsome ten; RNAi, RNA interference; ROS, reactive oxygen species; shRNAs, short hairpin RNAs; siRNAs, small interfering RNAs; TAMs, tumor‐associated macrophages; TET1, ten‐eleven translocation; TGF‐β2, tumor growth factor‐β2; VCR, vincristine.

### 5.1 Polyphenols

Dihydromyricetin, a flavonoid compound (belonging to polyphenols) extracted from the Japanese raisin tree (*Hovenia dulcis*), could inhibit both MRP2 expression and its promoter activity by inhibiting NF‐κB‐Nrf2 signaling in HCT116/L‐OHP and HCT‐8/VCR cell lines, contributing to the reversal of L‐OHP/VCR‐resistant CRC cells.^107^ Curcumin, as a lipophilic polyphenol, was effective in the inhibition of cell proliferation, increment of cell apoptosis, block of G0/G1 phase, and downregulation of 10 and 11 translocation (TET1, a DNA demethylase), and naked cuticle homolog 2 (NKD2, a negative regulator of Wnt signaling), suggesting that curcumin might exert antiresistance effect 5‐FU‐resistant HCT116 cells by modulating the TET1‐NKD2‐Wnt signaling pathway, thus inhibiting the EMT progress.^108^ Moreover, Fan et al. found that curcumin could evade tumor progression and reverse MDR in the HCT‐8/5‐FU cells line by suppressing the expression of P‐gp and heat shock protein‐27.^109^ Resveratrol, a naturally occurring polyphenol, could induce chemosensitization to 5‐FU in HCT116/5‐FU and SW480/5‐FU cell lines by inhibiting EMT via enhancing intercellular junctions and suppressing NF‐κB pathway.^110^ Resveratrol could also reverse L‐OHP resistance by increasing drug accumulation, inhibiting MDR1, NF‐κB pathway, and the transcriptional activity of cAMP‐responsive elements in L‐OHP‐resistant HCT116 cells.^111^

### 5.2 Terpenoids

β‐elemene, a sesquiterpene compound isolated from the Chinese herb *Curcumae Rhizoma*, could significantly inhibit cell proliferation and reverse the resistance of HCT116 p53^−/−−^ to 5‐FU by inducing cyclin D3‐dependent cycle arrest and autophagy.^112^ Atractylenolide II, a sesquiterpenoid monomer, which is extracted from traditional Chinese medicine *atractylodes macrocephala*, could inhibit tumor proliferation and increase chemosensitivity of Lovo, SW480 cell lines to 5‐FU, CDDP, mitomycin, and adriamycin.^113^

### 5.3 Quinones

Emodin, a natural anthraquinone derivative, could reverse 5‐FU resistance in SW480/5‐FU cells by blocking cell proliferation, invasion, and migration as well as increasing cell apoptosis and downregulating the PI3K/Akt pathway.^114^ Tanshinones, diterpene quinones isolated from the roots of *Salviamiltiorrhiza bunge*, owe the properties of antioxidation, anti‐inflammation, antitumor, and other pharmacological effects, and its chemoresistance reversal effect has been found in recent years.^115^ Tanshinone IIA, cryptotanshinone, and dihydrotanshinone are three of the compounds of tanshinones, which reverse chemoresistance to some extent in CRC. Tanshinone IIA could combat L‐OHP resistance in the SW480/L‐OHP cell line by the inhibition of the ERK/Akt signaling pathway. Tanshinone IIA applied with L‐OHP could significantly downregulate the expressions of Bcl‐2, p‐ERK, and p‐Akt, and upregulate the expressions of Bax and active caspase 3.^116^ Cryptotanshinone and dihydrotanshinone could prevent the growth of MDR‐resistant SW620/Ad300 cells by inducing autophagic cell death and p53‐independent cytotoxicity.^117^ Hypericin, a natural anthraquinone, is a well‐studied photosensitizer. Hypericin‐mediated photodynamic therapy could resensitize CRC‐resistant cells including HCT‐8/L‐OHP and HCT116/L‐OHP cell lines toward L‐OHP, which is associated with decreased drug efflux (downregulation of MRP2 level), GSH‐related detoxification, and nucleotide excision repair (NER)‐mediated DNA repair.^118^

### 5.4 Alkaloids

Evodiamine, an alkaloid, could inhibit cell growth and induce apoptosis and suppress ABCG2‐mediated MDR resistance in HCT116/L‐OHP cells by inhibiting p50/p65 NF‐κB pathway.^119^

### 5.5 Sterols

Ginsenoside Rh2 could inhibit cell proliferation and migration, induce apoptosis, and decrease the drug‐resistant correlated genes expression like MRP1, MDR1, lung resistance‐related protein (LRP), and glutathione S‐transferase (GST), which reverses chomoresistance in HCT‐8/5‐FU and LoVo/5‐FU cell lines.^120^ β‐Sitosterol, a phytosterol, could reverse L‐OHP MDR in HCT116/L‐OHP cells via breast cancer resistance protein (BCRP/ABCG2) suppression by disrupting murine double minute 2 (MDM2, an E3 ubiquitin ligase) binding to p53, thus evading protein degradation and ubiquitination, and resulting in the activation of p53 and apoptosis enhancement.^121^

### 5.6 Others

Salvianolic acid B, a phenolic acid isolated from the dried root and rhizome of *Salvia miltiorrhiza* Bge. (Labiatae), could reverse MDR in HCT‐8/VCR cells through elevating ROS levels, which promoted apoptosis and lowered the expression of P‐gp, which increased the chemosensitivity of drug‐resistant cancer cells.^122^

## 6 NEW DRUG DELIVERY SYSTEM

The low solubility and bioavailability, off‐target, and cytotoxicity to normal tissues of chemotherapy decreases the therapeutic effect and promotes drug resistance in CRC, which causes relapse and poor prognosis of CRC patients. To deal with it, a new drug delivery system including nanocarriers, liposomes, exosomes, and hydrogels has been used to overcome chemoresistance in CRC.

### 6.1 Nanocarriers

Lower toxicity and fewer side effects in blood and noncancerous tissues, increased aqueous solubility and bioavailability, specific target feature and multifunctional drug combination make nanoparticle‐based formulations become an effective strategy to overcome drug resistance in CRC combined with conventional chemotherapy.^123^ Chen et al reported that codelivery 5‐FU with epidermal growth factor (EGF) grafted hollow mesoporous silica nanoparticles (EGF‐HMSNs) can augment cytotoxicity and reverse MDR by enhancing drug accumulation in tumor cells, inducing S phase arrest and inducing cell death in 5‐FU‐resistant SW480 cells.^124^ Using aptamer‐conjugated grapefruit‐derived nanovectors (GNVs) loaded with DOX and P‐gp siRNA also showed MDR reversal effect, which could effectively inhibit proliferation and enhance apoptosis in MDR Lovo cells and the effect might be related to the downregulation of P‐gp.^125^ Other multifunctional codelivery systems, like mesoporous silica‐coated gold nanorods (GNRs/mSiO2) loaded with DOX, conjugated with pH‐responsive poly‐histidine (PHIS), and covered with d‐α‐tocopherol polyethylene glycol 1000 succinate (TPGS) was discovered to increase the drug accumulation and promote photothermal conversion in tumor resistant CRC cells and exert better anticancer effects in SW620/Ad300 xenograft mice without observed systemic toxicity compared with other chemotherapy or photothermal therapy alone.^126^ Combination use of sodium butyrate and Fe3O4 magnetic nanoparticles (MNPs), coated with folic acid (FA) and polyethylene glycol (PEG) (FA‐PEG@MNPs), could decrease cell viability and increase FA‐PEG@MNPs intracellular uptake in Lovo cells, which indicated that the combined use may exert MDR resistance reversal effect in CRC.^127^ Due to the multiple advantages of nanocarriers, recently, more studies are focusing on nanocarriers and trying to find different ways to overcome chemoresistance.^128^, ^129^

### 6.2 Liposomes

Liposomes, artificial colloidal vesicles with unique lipid bilayered membranes, can target tumor cells after chemical modification or physical treatment and have been used in the targeted therapy of cancer.^129^ Liposomes, as one kind of nanocarriers, own the advantages like biocompatibility and low systematic toxicity as well, which also contribute to drug resistance reversal. Xu et al found that bifunctional liposomes loaded with hypoxia‐inducible factor‐1(HIF‐1) inhibitors, acriflavine (ACF), and DOX (DOX‐ACF@Lipo) can reduce the chemoresistance to DOX in advanced CRC.^130^ Combining 5‐FU with a biochemical modulator in one same stealth liposome can exhibit significant antiproliferative activity and reverse drug resistance in resistant CRC cells.^131^ Liposome encapsulation of VCR and monoclonal antibody or verapamil can effectively overcome MDR in human colon cancer cells.^132^ Injectable PEGylated liposome encapsulating disulfiram was found to reverse chemoresistance at low nanomolar concentrations.^133^

### 6.3 Exosomes

Exosomes are endocytic extracellular vesicles (EVs) ranging from about 40–160 nm in diameter, which is associated with cancer proliferation, migration, invasion, and drug resistance.^134^, ^135^ Exomes induce drug resistance mainly by exporting chemical drugs from tumors or importing resistance‐associated small molecules to cancer cells. CRC‐derived exosomes have been found to enhance CRC chemoresistance by transferring cancer‐related miRNAs,^136^ lncRNAs,^137^ and circRNAs.^82^, ^138^ Due to their targeting specificity, high bioavailability, low immunogenicity, low toxicity, and good cell–cell communication capacity, exosomes have been employed as a new drug delivery system for drug resistance reversal in cancer treatment, including CRC.^139^ Exosomes can overcome chemoresistance by transferring therapeutic ncRNAs and chemical drugs. It was reported that engineered exosomes containing miR‐21i and 5‐FU can effectively overcome drug resistance and dramatically elevated the cytotoxicity of 5‐FU in the HCT‐116/5‐FU cell line, compared to the single use of either miR‐21i or 5‐FU.^34^ Exosomal miR‐204‐5p sustained released by human‐constructed HEK293T cells enhanced the 5‐FU sensitivity of LoVo and HCT116 cell lines, and inhibited neoplasm growth in HCT116 xenograft mice.^140^ Engineered exosomes loaded with lncRNA PGM5‐AS1 and L‐OHP realized by electroporation reversed L‐OHP resistance in DLD1/L‐OHP cell line and xenograft mice.^141^ Exosomes‐carried circ_0094343 improved the chemosensitivity to5‐FU, L‐OHP, and DOX via regulating the miR‐766‐5p/TRIM67 axis.^81^ Circular RNA F‐box and WD repeat domain containing 7 (circ‐FBXW7) encapsulated in exosomes by electroporation could enhance the chemosensitivity of SW480/L‐OHP and HCT116/L‐OHP cells to L‐OHP, increase apoptosis and inhibit EMT by binding to miR‐18b‐5p, the reversal effect was also found in the HCT116/L‐OHP xenograft mice.^142^ Kim et al. found that encapsulation of PTX to exosomes released from macrophages (exoPTX) by means of sonication could overcome MDR in resistant cancer cells by delivering the anticancer drug into the cancer cells directly, increasing drug accumulation and cytotoxicity, and bypassing the P‐gp‐mediated drug efflux. This effect was also verified in the murine model of lung metastases, exoPTX could significantly inhibit the tumor growth in the pulmonary. They also found that exosomes incorporated with doxorubicin (exoDOX) promoted the accumulation of DOX in resistant cancer cells.^143^ After a modification of PTX‐loaded exosomes with aminoethylanisamide‐polyethylene glycol (AA‐PEG) vector moiety, PTX exhibited more potent anticancer effect in lung metastasis compared to the single administration of exoPTX or PTX.^144^

### 6.4 Hydrogels

Hydrogels, an emerging drug carrier, are three‐dimensional (3D) frameworks comprised of cross‐linked hydrophilic polymers. Due to their excellent biocompatibility and biodegradability, hydrogels have been extensively utilized for the treatment of cancer chemoresistance and relapse in recent years.^145^ Hydrogels could modulate drug release when exposed to external stimuli including light, temperature, pH, and electrical and magnetic fields.^146^ It has been found that hydrogel carriers loaded with drugs could overcome chemoresistance in many cancers, mainly including breast cancer,^147^, ^148^, ^149^ ovarian cancer,^150^, ^151^ gastric cancer,^152^ and CRC.

5‐FU loaded in a thermal sensitive hydrogel system (a biodegradable PEG‐polycaprolactone (PCL)‐PEG (PECE) triblock copolymer), prolonged drug release time and markedly inhibited the growth and dissemination of CT26 cells in colorectal peritoneal carcinomatosis (CRPC) murine model.^153^ An alginate nanocomposite hydrogel coloaded with CDDP and gold nanoparticles (AuNPs), combining photothermal therapy with chemotherapy, dramatically inhibited the growth of 90% colorectal tumors of control and dramatically improve the animal survival studied in CT26 colorectal xenograft murine model. This chemophotothermal therapy based on hydrogel could directly transport high‐dose drugs and heat to tumors with neglectable side effects, which elevated the antitumor efficiency of CDDP and had the potential to evade CRC relapse.^154^ A thermo‐sensitive hydrogel coloaded with L‐OHP and tannic acid (TA) polymeric nanoparticles (L‐OHP/TA NPs), an injectable drug delivery system, could evade the growth of tumors and prolonged the survival time of the CT26 peritoneal CRC murine model.^155^ An injectable thermal‐sensitive poly(L‐glutamate)‐based hydrogel coloaded with CDDP and combretastatin A4 disodium phosphate (CA4P), exerted enhanced antitumor efficacy in C26 colon cancer‐bearing mice.^156^

## 7 COMBINATION THERAPY

Combination therapy is the common treatment for CRC. FOLFOX, capecitabine and oxaliplatin (CapeOX), and leucovorin, fluorouracil, and irinotecan hydrochloride (FOLFIRI) are the first‐line regimen for chemotherapy in CRC. Combination therapy could enhance anticancer efficacy, prolong survival, prevent drug resistance, and tumor relapse, which always makes an effect of “1 + 1 > 2.” Various combination therapies combating chemoresistance have been clarified in the upper five sections, so the combined therapies not belonging to these sections are illustrated below.

### 7.1 Combined with 5‐FU

Auranofin, a forkhead box 3 (FoxO3) agonist and thioredoxin reductase 1 (TR1) inhibitor, could enhance the sensitivity of HCT‐8/5‐FU and SW620/5‐FU cells to 5‐FU in vitro and the sensitivity of SW620/5‐FU cells to 5‐FU treatment in vivo by inhibiting the nuclear factor erythroid 2‐related factor 2 (Nrf2)/TR1 signaling pathway. Auranofin in combination with 5‐FU induced the most cell death compared to treatment with auranofin or 5‐FU alone and suppressed tumor cell invasion obviously. In SW620/5‐FU cells xenograft nude mice, fewer lung metastatic nodules were observed in auranofin plus 5‐FU group than in the 5‐FU or auranofin groups.^157^ It has been found that *Lactobacillus Plantarum* Supernatant (LP SN) combined with 5‐FU could reverse the resistance to 5‐FU and enhance the therapeutic effect of 5‐FU in 5‐FU‐resistant HT29 cells and HCT116 cells by inactivating the Wnt/β‐catenin signaling, reducing CSCs, and increasing apoptosis.^158^ Curaxins, a class of small molecules, might overcome 5‐FU resistance in defective mismatch‐repair (dMMR) colorectal cancer patients by targeting the histone chaperone facilitates chromatin transcription (FACT) complex.^159^

### 7.2 Combined with L‐OHP

Administration of Toll‐like receptor agonists R848 combined with L‐OHP reversed the functional orientation of myeloid‐derived suppressor cells (MDSCs) toward tumoricidal M1‐like macrophages and strengthened the antitumor effect of L‐OHP.^160^ Combinational inhibition of YAP by a YAP‐specific inhibitor verteporfin and an inhibitor of EGFR/ErbB1 AG1478 synergistically inhibited the CRC progression and overcame chemoresistance.^161^ GW4064, a synthetic farnesoid X receptor (FXR) agonist, could enhance the antineoplasm effects of L‐OHP by inducing BAX/caspase‐3/gasdermin E (GSDME)‐mediated pyroptosis in HT26 and SW620 cells.^162^

### 7.3 Combined with DOX

Tamoxifen, a selective estrogen receptor modulator, combined with DOX can reverse MDR in CRC nude mice, reduced weight and volume of transplanted tumors, which is independent of the expression of estrogen receptors and has no influence on MDR1 expression.^163^

## 8 CONCLUSION

CRC is one of the most prevalent cancers across the world with increasing incidence in recent years, and the chemoresistance caused by long‐term use has evaded the therapeutic process of CRC. Chemical drugs, combined with clinical common drugs, herbal medicine monomers, gene therapy, and protein inhibitors, could restore the chemosensitivity of tumors to chemodrugs. Nanocarriers, liposomes, exosomes, and hydrogels, as drug delivery systems, could combine multiple drugs together well, transport these drugs to the tumor site directly, and control the drug release, which dramatically enhances the drug effect and reverse the drug resistance occurred in mono‐drug therapy. Reversal drugs involved in drug repurposing and herbal medicinal compound are already marketed drugs, and are the safest and fastest mean able to apply in the clinic, thus more efforts should be contributed in these fields. Gene therapy and protein inhibitors, belonging to gene and protein levels, respectively, become a hot spot in cancer treatment and chemoresistance. Moreover, gene therapy in chemoresistance reversal still stays in the early stage but own huge developmental potential. So far, although various potential strategies have been found, there is still a long way to go from laboratory to clinic. Therefore, further and consistent investigation is required to the realization of the victory in the chemoresistance combat of CRC.

## AUTHOR CONTRIBUTIONS

**Shuchang Ma:** Visualization (lead); writing – original draft (lead); writing – review and editing (lead). **Jiaqi Zhang:** Visualization (lead); writing – review and editing (lead). **Tianhua Yan:** Writing – review and editing (equal). **Mingxing Miao:** Writing – review and editing (equal). **Yemin Cao:** Writing – review and editing (equal). **Yongbing Cao:** Writing – review and editing (equal). **Lichao Zhang:** Writing – review and editing (equal). **Ling Li:** Supervision (lead); validation (lead); visualization (lead); writing – review and editing (lead).

## FUNDING INFORMATION

This study was supported by grants from the National Natural Science Foundation of China (No. 81973551), the Science and Technology Commission of Shanghai Municipality (No. 19ZR1451800, 21ZR1460400), the Innovative projects of Shanghai University of Traditional Chinese Medicine (Y2021030), the Future plan for Traditional Chinese Medicine development ofScience, the Health Commission of Shanghai Municipality (ZY(2021‐2023)‐0203‐04) and the Technology of Shanghai Municipal Hospital of Traditional Chinese Medicine (WL‐HBBD‐2021001 K).

## CONFLICT OF INTEREST

The authors declare no potential conflict of interest.

## Data Availability

Data sharing is not applicable to this article as no new data were created or analyzed in this study.
